# Intraoperative Brain Mapping by Cortico-Cortical Evoked Potential

**DOI:** 10.3389/fnhum.2021.635453

**Published:** 2021-02-18

**Authors:** Yukihiro Yamao, Riki Matsumoto, Takayuki Kikuchi, Kazumichi Yoshida, Takeharu Kunieda, Susumu Miyamoto

**Affiliations:** ^1^Department of Neurosurgery, Kyoto University Graduate School of Medicine, Kyoto, Japan; ^2^Division of Neurology, Kobe University Graduate School of Medicine, Kobe, Japan; ^3^Department of Neurosurgery, Ehime University Graduate School of Medicine, Toon, Japan

**Keywords:** cortico-cortical evoked potential, intraoperative monitoring, large-scale cortical network, brain function, awake craniotomy, electrical stimulation, brain mapping

## Abstract

To preserve postoperative brain function, it is important for neurosurgeons to fully understand the brain's structure, vasculature, and function. Intraoperative high-frequency electrical stimulation during awake craniotomy is the gold standard for mapping the function of the cortices and white matter; however, this method can only map the “focal” functions and cannot monitor large-scale cortical networks in real-time. Recently, an *in vivo* electrophysiological method using cortico-cortical evoked potentials (CCEPs) induced by single-pulse electrical cortical stimulation has been developed in an extraoperative setting. By using the CCEP connectivity pattern intraoperatively, mapping and real-time monitoring of the dorsal language pathway is available. This intraoperative CCEP method also allows for mapping of the frontal aslant tract, another language pathway, and detection of connectivity between the primary and supplementary motor areas in the frontal lobe network. Intraoperative CCEP mapping has also demonstrated connectivity between the frontal and temporal lobes, likely *via* the ventral language pathway. Establishing intraoperative electrophysiological monitoring is clinically useful for preserving brain function, even under general anesthesia. This CCEP technique demonstrates potential clinical applications for mapping and monitoring large-scale cortical networks.

## Introduction

To preserve brain function postoperatively, it is necessary to identify and have a thorough understanding of the neural connectivity between cortical eloquent areas. This is important to help neurosurgeons in maximally preserving brain function when surgically treating brain lesions.

Intraoperative high-frequency direct electrical stimulation (DES) is a popular method to detect eloquent areas during brain resection. During awake craniotomy, DES introduces a biphasic constant-current stimulation (50–60 Hz) whilst optimizing intraoperative tasks (e.g., motor or language tasks) (Berger et al., 1989; Silbergeld et al., 1992). DES can map the eloquent cortices related to language or higher cognitive functions. The “eloquent” white matter pathways (e.g., association fibers related to language or motor functions) have been extensively identified (Duffau et al., 2005); however, the mechanisms through which DES works are poorly understood, particularly in terms of cortical spreading. Moreover, DES can only map stimulus site regions during awake craniotomy and can cause seizures. Therefore, additional methods are required to detect brain function intraoperatively.

Other methods have recently been developed to probe *in vivo* brain connectivity, including anatomical and functional connectivity. Anatomical connectivity can be described by magnetic resonance imaging (MRI)-based diffusion tractography. Diffusion tractography allows the visualization of *in vivo* large white matter pathways (Catani et al., 2002; Mori and Van Zijl, 2002). This non-invasive technique is widely used for preoperative and intraoperative evaluations, aimed at tracing white matter pathways, which are related to eloquent brain functions; however, these fibers are solely identified by the reflection of anisotropic diffusion of their water molecules through tractography, suggesting that these tracts may not necessarily have the associated function. Combination of tractography with electrophysiological methods such as DES complement each other and help to elucidate brain function.

As an electrophysiological method of probing functional brain connectivity, we review the efficacy of cortico-cortical evoked potentials (CCEPs) in mapping functional brain networks and evaluate its clinical utility in preserving brain function intraoperatively.

## CCEP Method

### Procedure

The CCEP technique was originally introduced as an extra-operative procedure preceding epilepsy surgery (Matsumoto et al., 2004, 2007). To prevent possible seizure induction, CCEP methods are performed after antiepileptic medications are administered. Single-pulse (1 Hz) electrical stimulation (ES) is applied to the cortex, and CCEPs are recorded from functionally connected cortices. The ES comprises a constant-current pulse (square-wave pulse width of 0.1–1 ms) at a fixed frequency of 1 Hz (0.2, 0.5, or 2 Hz), delivered in a bipolar fashion through a pair of adjacent electrodes placed on the cortices. In our intraoperative setting, the intensity is set at 10–15 mA (monophasic square wave pulse, alternating polarity, 0.3 ms duration). An electrocorticogram is recorded at a 1,000–5,000 Hz sampling rate, with the low-frequency filter set at 0.08–1 Hz. The reference electrodes of all subdural electrodes are placed on the skin over the mastoid process of the side contralateral to the implanted electrodes. CCEPs are obtained by averaging the electrocorticogram with a time window from −100 to 500 ms, time-locked to the stimulus. In each session, to confirm the reproducibility of each response, two or three trials of 20–30 stimulations are averaged independently.

CCEP generally comprises an early sharp negative potential (N1: peak latency 10–50 ms) and a consecutive slow negative potential (N2: peak latency 50–300 ms) (Matsumoto et al., 2004; Yamao et al., 2014). An N1 response can occasionally be recorded as a positive potential probably because it represents the positive end of the dipolar activity in the recorded sulcus (Matsumoto et al., 2004; Terada et al., 2008).

### General Mechanism of CCEP

**Figure 1:**
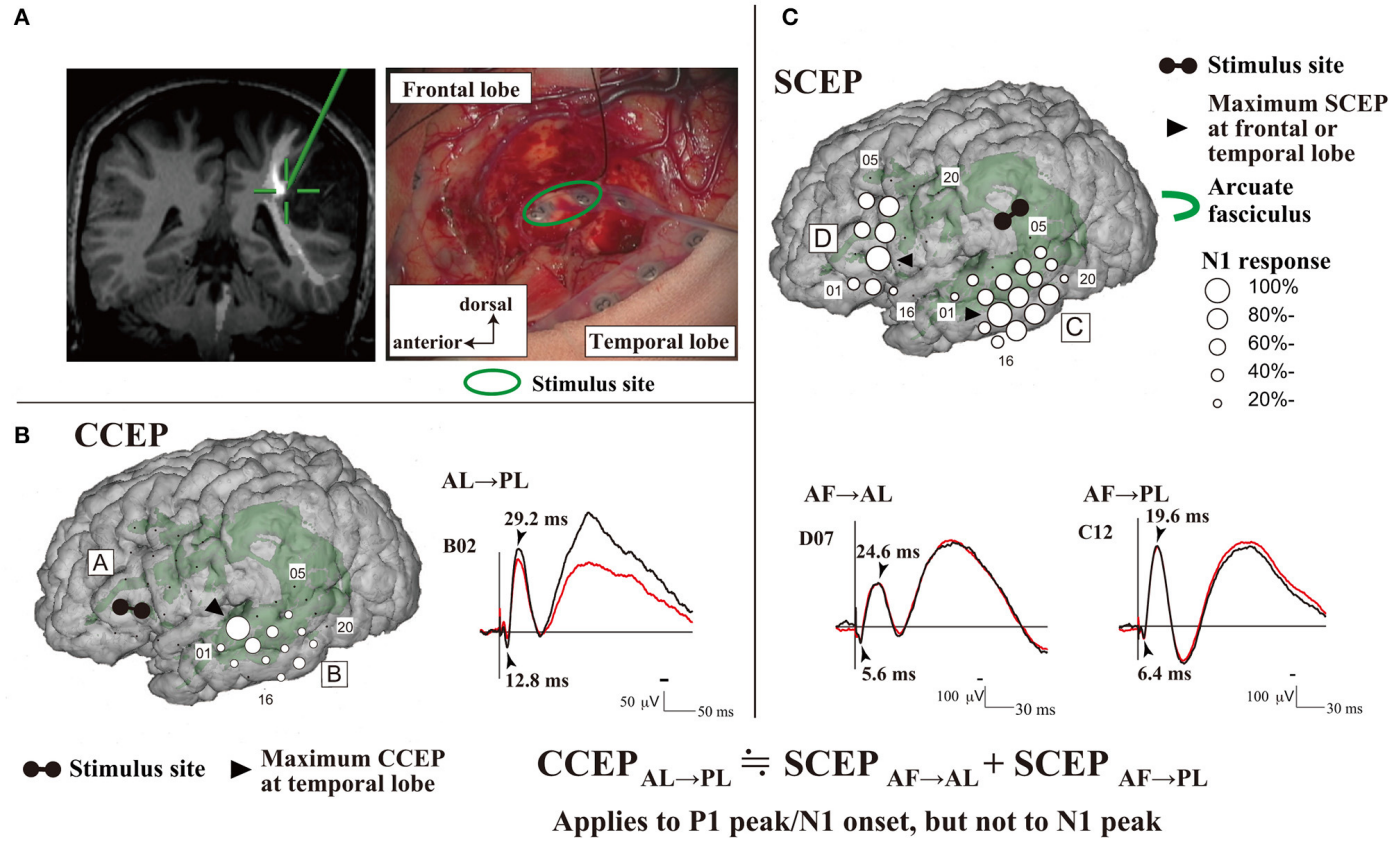
Intraoperative CCEPs and SCEPs. **(A)** The schema of stimulus site (electrode pair, green circle) at the floor of the tumor resection cavity is shown. The stimulus site (green cross) was closely attached to the AF. **(B)** Single-pulse electrical stimulation of the AL produced CCEPs in the temporal lobe. The diameter of the circle at each electrode represents the percentile to the largest amplitude (N1) at the maximum CCEP response site. **(C)** Single-pulse electrical stimulation was applied to the stimulus site (AF), and SCEPs were recorded both from the anterior language area (SCEP_AF→AL_, D plate) and posterior language area (SCEP_AF→PL_, C plate) at and around the terminations of the AF tract. The diameter of the circle at each electrode represents the percentile to the largest amplitude (N1) at the each maximum SCEP_AF→AL_ or SCEP_AF→PL_ response site, respectively. Notably, at the maximum response sites, the summation of P1 peak/N1 onset latencies of SCEPs (SCEP_AF→AL_ + SCEP_AF→PL_, 12.0ms) was close to the P1 peak/N1 onset latency of CCEP_AL→PL_ (12.8ms). AF, arcuate fasciculus; AL, anterior language area; CCEP, cortico-cortical evoked potential; PL, posterior language area; SCEP, subcortico-cortical evoked potential. Adapted with permission from Yamao et al. (2014).

The mechanism underlying CCEPs remains unclear. Two mechanisms of impulse propagation have been suggested: (1) cortico-cortical propagation is directly conveyed through white matter fibers; and (2) cortico-subcortico-cortical propagation is indirectly conducted *via* subcortical structures, such as thalamus. A previous CCEP study investigating the parieto-frontal connectivity revealed a linear correlation between the N1 peak latencies and distance from the stimulus to the CCEP response sites (Matsumoto et al., 2012). This supports a direct cortico-cortical pathway rather than an indirect cortico-subcortico-cortical circuit, since the longer the surface distance is, the longer the actual white matter pathway (i.e., conducting time) connecting the two cortical sites is. This hypothesis has been further supported by an intraoperative study that demonstrated CCEP connectivity can be recorded between the anterior (AL) and posterior language areas (PL) (Yamao et al., 2014). Additionally, subcortico-cortical evoked potentials were also recorded from both the AL and PL by single-pulse ES to the arcuate fasciculus (AF) at the floor of the resection cavity. Comparisons between subcortico-cortical evoked potential and CCEP latencies revealed that the first volley from the stimulation, namely the N1 onset, was directly conveyed through the white matter pathway (i.e., the AF; Figure 1). The N1 onset response (approximate latency ≤10 ms) may represent the fastest monosynaptic impulse conveying the middle or deep cortical layers by the projection of cortico-cortical fibers (Terada et al., 2012). The N2 response may be generated from the surrounding cortex *via* either a local cortico-cortical projection or a cortico-subcortico-cortical reverberating circuit (Matsumoto et al., 2004, 2017).

## Intraoperative Probing of Functional Networks by CCEPs

Extraoperatively, CCEPs are clinically used to probe functional networks and seizure propagation to identify epileptogenicity *via* subdural or depth electrodes (Matsumoto et al., 2004, 2007, 2017; Koubeissi et al., 2012; Enatsu et al., 2013b, 2015; Matsuzaki et al., 2013; Keller et al., 2014; Mitsuhashi et al., 2020, 2021). CCEP recording is highly practical for various reasons, including that patients are not required to perform any specific task during stimulation and can simply lie on a bed. Moreover, cortico-cortical connectivity can be probed from one stimulus site within 1 min, and seizure induction is extremely rare (0.39% reported in a recent CCEP study) (Kobayashi et al., 2021). Because of the marked practicality and feasibility, CCEP monitoring is now also used in intraoperative settings to probe and monitor functional brain networks, such as those involved in language and motor functions (Kikuchi et al., 2012; Saito et al., 2014; Yamao et al., 2014, 2017b; Enatsu et al., 2016; Tamura et al., 2016; Ookawa et al., 2017; Kanno et al., 2018; Suzuki et al., 2019; Yoshimoto et al., 2019; Nakae et al., 2020; Shibata et al., 2020; Vincent et al., 2020). CCEP monitoring is also able to detect connection or disconnection of an epileptogenic network during epilepsy surgery (Inoue et al., 2018; Kamada et al., 2018, 2020) (Table 1).

**Table 1:** Intraoperative CCEP and SCEP studies.

**TARGET FIBERS**	
**Language network**	
Dorsal pathway	Saito et al., 2014; Yamao et al., 2014, 2017b; Enatsu et al., 2016; Tamura et al., 2016; Kanno et al., 2018; Suzuki et al., 2019; Vincent et al., 2020
Ventral pathway	Nakae et al., 2020
Frontal aslant tract	Ookawa et al., 2017
**Motor network**	
Pyramidal tract	Enatsu et al., 2016
Short U fibers	Kikuchi et al., 2012; Shibata et al., 2020; Vincent et al., 2020
**Epileptogenic network**	Inoue et al., 2018; Kamada et al., 2018

*CCEP, cortico-cortical evoked potential; SCEP, subcortico-cortical evoked potential*.

### Language Function

A dual-stream model for language processing has been recently proposed (Hickok and Poeppel, 2004). The dorsal pathway carries auditory–motor integration by mapping acoustic speech sounds to the articulatory representations, while the ventral pathway serves as a sound-to-meaning interface. Recent tractography and DES studies have demonstrated that the dorsal and ventral pathways primarily involve the AF or superior longitudinal fasciculus, and the uncinate (UF), inferior longitudinal, or inferior fronto-occipital fasciculus (IFOF), respectively (Catani et al., 2005; Duffau et al., 2005).

Intraoperative online sequential recording of motor evoked potentials (MEPs) and somatosensory evoked potentials (SEPs) can map the motor cortices and output pathway (cortico-spinal tract), and the sensory cortices and ascending pathway, respectively, under general anesthesia (Macdonald et al., 2013, 2019). Contrastingly, no intraoperative electrophysiological methods have been established for real-time monitoring of language function under general anesthesia. We recently applied intraoperative CCEPs for intraoperative monitoring of the dorsal language white matter pathway (the AF) mainly in awake patients (Yamao et al., 2014, 2017b), in the following sequence (Figure 1):

(1) Subdural electrodes were covered on the ventrolateral frontal and lateral temporoparietal cortices, which were localized based on anatomical criteria or by using preoperative neuroimaging studies (i.e., language-tasked functional MRI and/or diffusion tractography).

(2) Single-pulse ES was applied to the ventrolateral frontal cortices. Based on the CCEP distribution (the largest CCEP connectivity) in the lateral temporoparietal cortices, we determined the stimulus site (the putative AL) and recording site (the putative PL).

(3) To evaluate the integrity of the dorsal language pathway, online sequential CCEP_AL→*PL*_ (stimulating the AL and recording CCEPs from the PL) monitoring was performed under general anesthesia, and in the awake condition by measuring the N1 amplitude at the largest CCEP response site.

(4) When available, DES was also applied only to the AL to confirm the language function, due to the time limitation in the intraoperative setting. To identify the reciprocal connection between the AL and PL, single-pulse ES was also applied to the electrode in the lateral temporoparietal area, and CCEP_PL→*AL*_ was recorded.

The anatomical AL is localized, and projection from the PL to AL is more convergent. Thus, the connection between the two areas appears to be bidirectional, and CCEP_AL→*PL*_ responses are observed to have a larger distribution than CCEP_PL→*AL*_ responses (Matsumoto et al., 2004). Contrarily, the PL areas have a wide distribution, and their functional shifts can affect the surrounding cortices (Enatsu et al., 2013a). Thus, the CCEP_AL→*PL*_ was performed to monitor the dorsal language pathway, and the frontal stimulus site was confirmed as the core AL by using DES in all available cases (Yamao et al., 2017b). By combining 50 Hz and 1 Hz cortical and subcortical ES, we demonstrated that CCEP connectivity could map the AL and PL, even intraoperatively, and that the eloquent subcortical fiber (AF) provided a direct electrophysiological connection to the cortices (AL and PL). Moreover, we found that intraoperative dorsal language network monitoring may be feasible even under general anesthesia or even without full preoperative neuroimaging. CCEP_PL→*AL*_ responses were successfully recorded from the frontal stimulus site of CCEP_AL→*PL*_ in all available cases (Yamao et al., 2017b). Intraoperative dorsal language pathway monitoring by CCEP_PL→*AL*_ was available for tumors in certain locations (Saito et al., 2014; Yamao et al., 2017b).

**Figure 2:**
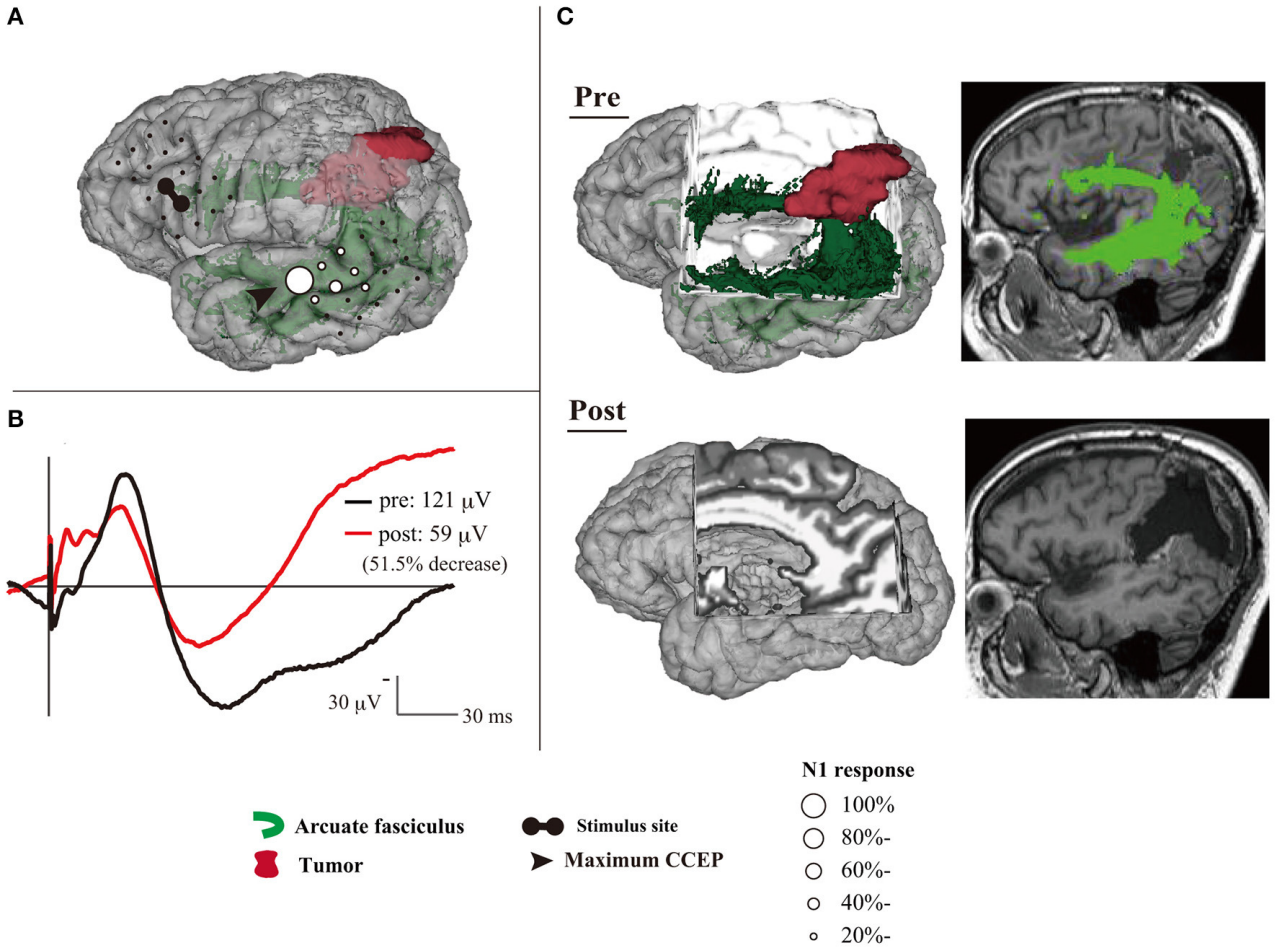
An illustrative case with > 50% decrease of CCEP_AL→*PL*_ N1 amplitude during tumor removal. **(A)** Three-dimensional MRI shows CCEP distribution under general anesthesia, using a circle map. **(B)** The change of N1 waveforms at the maximum CCEP_AL→*PL*_ response site are described. The black line shows the N1 waveform before the tumor removal, and the red line represents the N1 after tumor removal. The N1 amplitude decreased by 51.5% during the tumor removal. **(C)** Two- and three-dimensional MRI reveals that the postoperative AF tract became untraceable. The patient developed further phonemic paraphasia and impairment of repetition after surgery; these symptoms continued until the final follow-up. AF, arcuate fasciculus; CCEP, cortico-cortical evoked potential; MRI, magnetic resonance imaging. Adapted with permission from Yamao et al. (2017b).

CCEP has the potential for clinical applications in intraoperative mapping and monitoring of the language network. Based on a small cohort, patients with a 50% N1 decrease only suffer transient language dysfunction; however, one patient with a 51.5% N1 decrease suffered postoperative language dysfunction until their final follow-up, which is likely due to damage to the AF (Yamao et al., 2017b) (Figure 2). Although further case accumulation is warranted, this suggests the cut-off value for preserving the dorsal language pathway might be a 50% decrease in the CCEP N1 amplitude, as MEPs and SEPs (Macdonald, 2006; Macdonald et al., 2019; Saito et al., 2019). CCEP amplitudes are increased in the awake condition compared to the general anesthesia condition (Suzuki et al., 2019). Moreover, CCEP distribution, including the maximum CCEP response site, does not change (Yamao et al., 2017a). Even during vascular surgery under general anesthesia, CCEP monitoring is feasible, and it is sensitive to ischemic change, compared to MEPs or SEPs (Yoshimoto et al., 2019). Thus, the CCEP method enables intraoperative monitoring to preserve the dorsal language pathway, even under general anesthesia.

**Figure 3:**
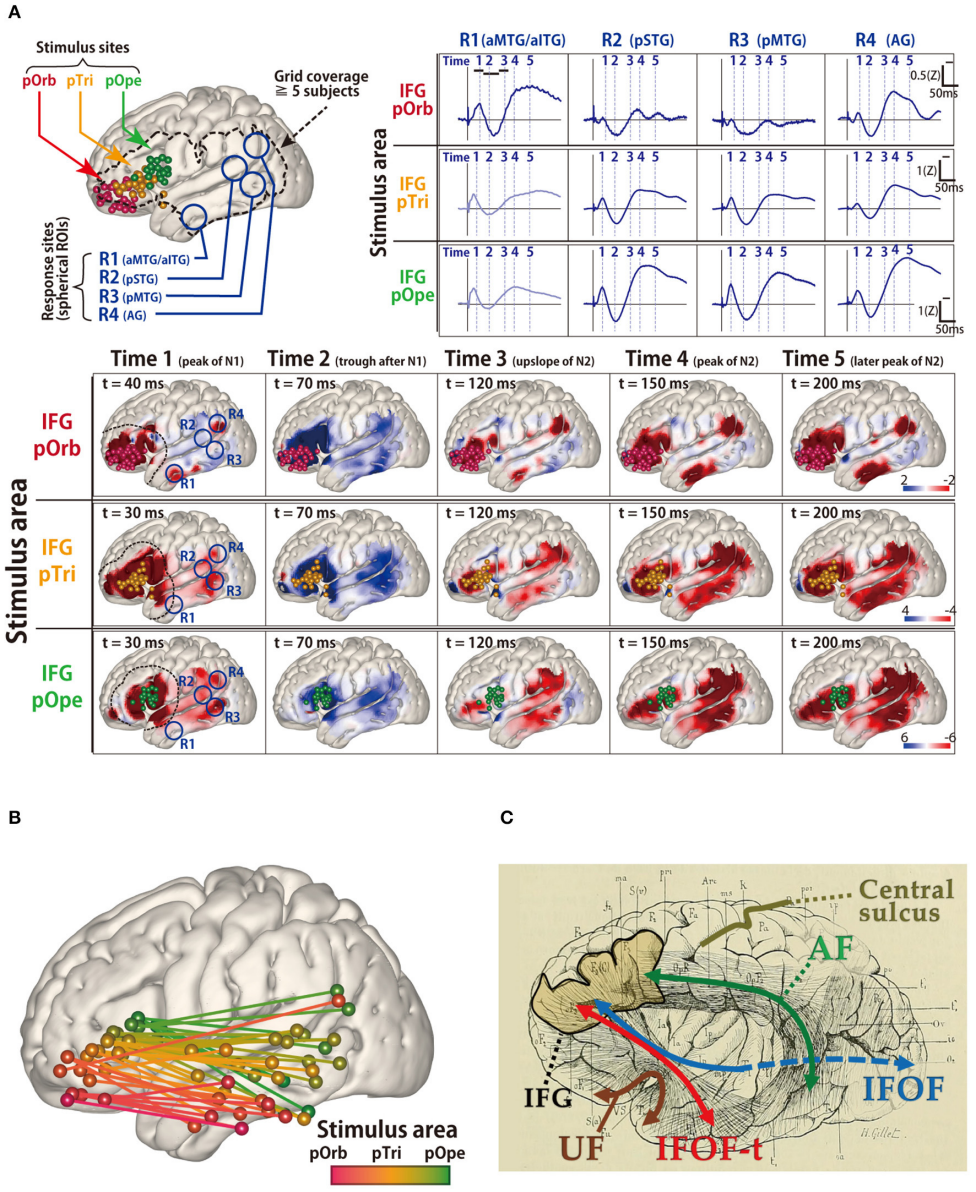
Probing of the perisylvian language pathway by intraoperative cortico-cortical evoked potential (CCEP). **(A)** The CCEP response map at the temporal lobe in 14 patients. (Upper left) MRI shows all stimulus sites in the inferior frontal gyrus (IFG), which are transferred to Montreal Neurosciences Institute space with spheres (red, pOrb; yellow, pTri; green, pOpe). The dotted line represents a recording area. (Upper right) The representative waveforms of the response sites in the four ROIs. The four ROIs were located as follows: R1, anterior parts of the MTG/ITG; R2, posterior part of the STG; R3, posterior part of the MTG; and R4, the AG. (Lower) The table shows the averaged response maps in the time sequence stratified by IFG subdivision. The five time points were set (Time 1; the peak of N1, Time 2; the trough after N1, Time 3; the upslope of N2, Time 4; the peak of N2, and Time 5; the peak of the latest N2). Electrical stimulation of the pOpe elicited CCEP responses widely in the temporoparietal area, while electrical stimulation of the pOrb responses in the anterior part of the MTG and IFG, and AG. By electrical stimulation of the pTri, a mixture of the patterns for the two stimulus areas is elicited. (Upper right) The representative waveforms of the response sites in the four ROIs. The four ROIs were located as follows: R1, the aMTG/aITG; R2, the pSTG; R3, the pMTG; and R4, the AG. **(B)** The schema of the connectivity between the IFG and lateral aspect of temporal lobe detected by CCEP. The gradation from red to green colors shows the transfer from the anterior to the posterior stimulation site at the IFG. **(C)** The schema of the white matter pathways in the vicinity of the IFG, overlaid on the reprinted schema of dissection from the classical textbook “Anatomie des centers nerveux” (Dejerine and Dejerine-Klumpke, 1895). A fan-shaped fiber is shown which connects the frontal lobe to the anterior part of the temporal lobe through the temporal stem, which is newly referred to as the temporal branch of the IFOF (IFOF-t). AG, angular gyrus; CCEP, cortico-cortical evoked potential; IFOF, inferior fronto-occipital fasciculus; ITG, inferior temporal gyrus; MRI, magnetic resonance imaging; MTG, middle temporal gyrus; pOpe, pars opecularis; pOrb, pars orbitalis; pTri, pars triangularis; ROI, region of interest; STG, superior temporal gyrus. Adapted with permission from Nakae et al. (2020).

The ventral language pathway has been reported to involve the UF, inferior longitudinal fasciculus, or IFOF (Duffau et al., 2005); however, in previous dissection and tractography studies (Catani et al., 2002; Martino et al., 2011), the cortical terminations of these three fibers did not reach the lateral temporal aspects (i.e., the PL). DES of the UF does not disturb object naming, and thus, resection of the UF is considered acceptable (Duffau et al., 2009). Therefore, the fibers involved in the ventral pathway remain unidentified. In a recent intraoperative CCEP study (Nakae et al., 2020), the pars opercularis of the inferior frontal gyrus (IFG) is connected to the posterior temporal cortices and supramarginal gyrus, whereas the pars orbitalis is connected to the anterior lateral temporal cortices and angular gyrus. The different connectivity of each IFG subdivision to the temporal lobe can result in an anterior–posterior gradient connectivity map (Figures 3A,B). The connection between the pars orbitalis of the IFG and anterior lateral temporal lobe implies the existence of a temporal branch of the IFOF, which is referred to as the IFOF-t (Figure 3C). The clinical implications of this branch remain unclear, and therefore, further ES studies investigating it are necessary.

Recently, a new language-related white matter fiber pathway has been described in the frontal lobe (Catani et al., 2012). This pathway, the frontal aslant tract (FAT), runs between the supplementary motor area (SMA) and the pars opercularis of the IFG (Catani et al., 2012; Vergani et al., 2014). A DES study revealed that the FAT is associated with verbal fluency as part of the negative motor network (Kinoshita et al., 2015). Therefore, its preservation is also required to prevent postoperative language dysfunction. In another recent study (Ookawa et al., 2017), intraoperative CCEPs were found to describe a cortico-cortical network between the IFG and superior frontal gyrus *via* the FAT, in a reciprocal manner. Thus, the CCEP method also allows for intraoperative monitoring of the FAT.

The dynamics of synchronous or oscillatory patterns of neuronal activity recorded on electrocorticogram have been recently proposed as potential neurophysiological factors of cortical processes (Salinas and Sejnowski, 2001). Notably, high gamma activity, ranging between 60 and 140 Hz, is considered to reflect localized cortical processing, such as language function (Crone et al., 2001). “Passive mapping” by combining high gamma activity and CCEP has been proposed (Tamura et al., 2016). First, real-time high gamma activity mapping of the PL is performed by listening to linguistic sounds. Second, 1 Hz ES is delivered to the identified PL to detect CCEPs in the frontal lobe (i.e., the AL). Then, the functioning of the AL and PL are confirmed by DES. The sensitivity and specificity of this passive mapping method are reported to be 93.8 and 89%, respectively. This passive mapping technique allows for neurosurgeons to create a new method of brain mapping using CCEPs.

Although additional studies are needed to establish intraoperative monitoring methods, CCEP is an efficient intraoperative method for mapping and preserving language function.

### Motor Function

The SMA is an eloquent brain region that controls complex motor functions, such as planning, initiation, learning, and language function, particularly in the dominant hemisphere (Vergani et al., 2014). Surgical resection of the SMA causes contralateral akinesia, mutism or speech disturbances, and difficulties with alternating hand movements, which is referred to as SMA syndrome (Laplane et al., 1977). These symptoms tend to resolve in several weeks, but are occasionally permanent (Krainik et al., 2001). The severity of the symptoms is related to the extent of SMA resection (Fontaine et al., 2002). Therefore, intraoperative mapping and preservation of the SMA are necessary for preserving postoperative motor function, or to minimize the severity of symptoms, analogous to preserving the primary motor area (M1).

**Figure 4:**
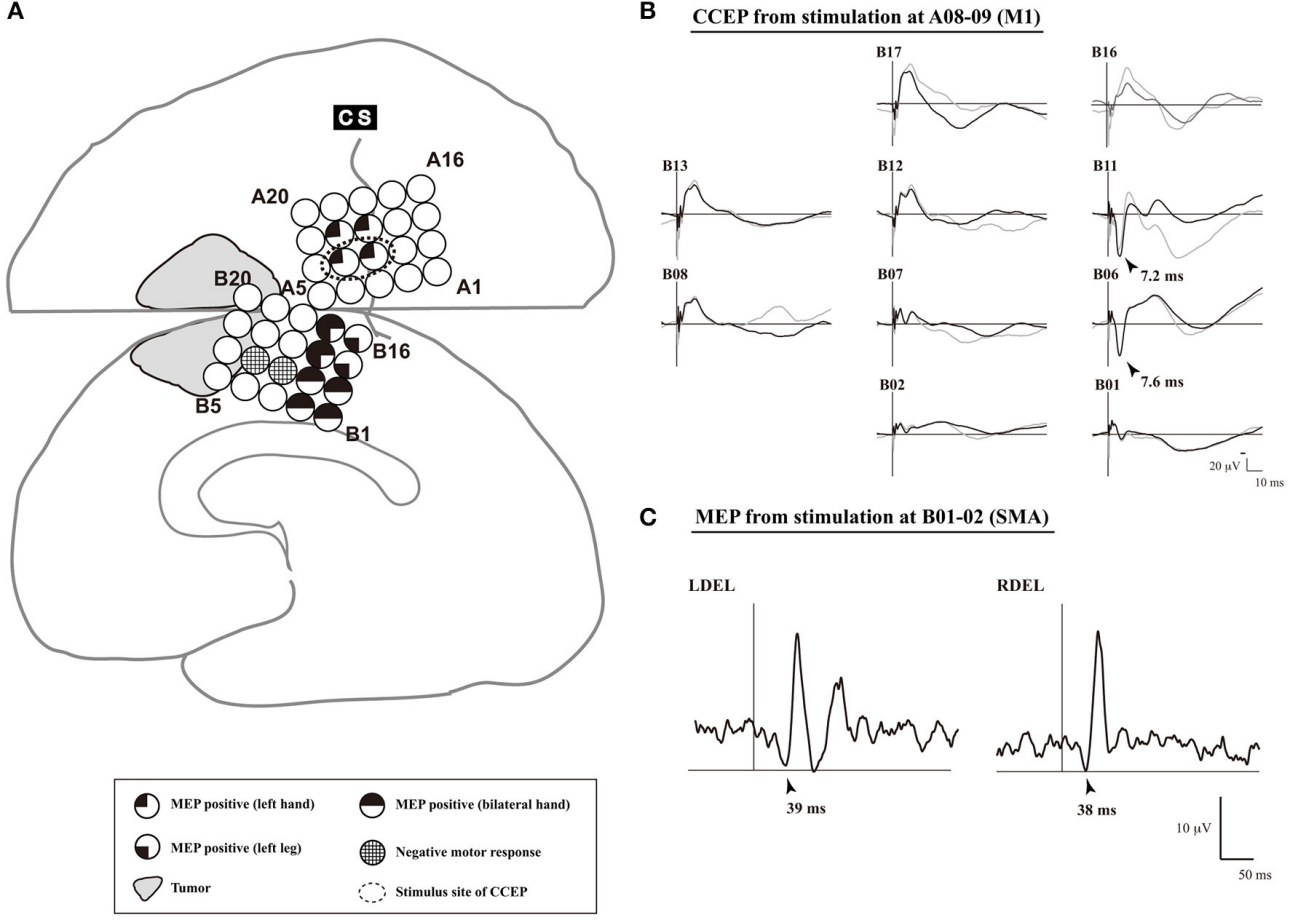
An illustrative case of SMA mapping. **(A)** The schema of cortical mapping by MEPs. Subdural grid electrodes are placed on the right lateral frontoparietal and medial frontal cortices. Single pulse electrical stimulation reveals positive motor functions based on MEPs. In electrodes B08–09, a negative motor response is elicited by 5-train electrical stimulation. **(B)** CCEP waveforms (Plate B). Single-pulse electrical stimulation was applied to a pair of electrodes A08–09 (Plate A), which was defined as the primary motor area of the left hand. Two trials are plotted in superimposition. A large CCEP response was recorded in electrodes B06, B11-13, and B17. The functional brain region associated with those electrodes was confirmed as the SMA by MEP. **(C)** MEP waveforms (electrodes B06–07). MEP was elicited from the bilateral deltoid muscles, and the site of electrodes B06-07 was defined as the SMA. CCEP, cortico-cortical evoked potential; CS, central sulcus; DEL, deltoid muscle; L, left; MEP, motor evoked potential; R, right; SMA, supplementary motor area. Adapted with permission from Shibata et al. (2020) and Yamao et al. (2015).

The SMA is divided into two functional subdivisions: the pre-SMA and SMA proper. The pre-SMA is related to higher-order aspects of motor control when performing motor tasks, whereas the SMA proper is activated during pure motor function (Picard and Strick, 1996). A previous dissection and tractography study (Vergani et al., 2014) demonstrates that the SMA-proper is connected to the precentral gyrus *via* short U-fibers connecting neighboring gyri. In an extra-operative setting, cortico-cortical functional connections between the lateral motor cortex (including the M1) and the medial motor cortex (including the SMA) are detected using CCEPs (Matsumoto et al., 2007). Additionally, studies have investigated the electrophysiological mapping of the SMA in intra- or extra-operative settings (Usui et al., 2008; Kikuchi et al., 2012). These studies found that a single-pulse or trains of five high-frequency ES of the SMA proper elicits MEPs with a longer latency than does a similar stimulation of the M1. Mapping using implanted subdural electrodes in an extra-operative setting requires two-stage surgery. Therefore, to reduce the burden on patients, intraoperative mapping of the SMA proper by means of CCEP connectivity in the following sequence was recently proposed (Yamao et al., 2015; Shibata et al., 2020) (Figure 4):

1. Under general anesthesia, subdural electrodes are placed on the lateral frontoparietal and medial frontal cortices to cover the anatomical M1 and SMA.
2. After recording the SEP obtained with median nerve stimulation, we apply a 5-train ES (with a pulse width of 0.2–0.3 ms at a frequency of 500 Hz, in a bipolar fashion) to the candidate electrodes through a pair of adjacent electrodes, and record the MEP of the contralateral extensor carpi radialis or first dorsal interosseous to detect the M1.
3. Single-pulse ES is applied to the detected M1, and CCEPs are recorded from the medial frontal cortices, to probe the cortico-cortical connections between the M1 and the SMA proper. The SMA proper is defined by distinguished CCEP N1 responses in the medial frontal area. A 5-train ES is delivered to the estimated SMA proper, to confirm the SMA proper by recording MEPs from the upper and lower extremities.

In a previous study, patients with preservation of the identified SMA proper did not display any new neurological deficits during or after surgery (Shibata et al., 2020). Thus, intraoperative CCEP monitoring can be clinically useful for mapping the SMA proper and preserving permanent motor deficits due to disturbance of the SMA proper. Further case accumulation is necessary to establish methods that can be used even under general anesthesia.

## Limitations

Intraoperative CCEP has some limitations. First, the data were obtained from patients with tumors or epilepsy. Their backgrounds, with regard to drugs, including antiepileptic drugs, location and pathology of the tumors, the grid coverage areas, and stimulus intensities varied across studies, and could have affected the CCEP results. Second, the subdural electrodes used have limitations in terms of spatial resolution and exploration of the cortical sulci (Ookawa et al., 2017). In addition, CCEP data were available only within the coverage area (i.e., the electrode placement area). Due to the lack of information outside this limited area, there may be functional connections other than those detected by CCEP. Third, the mechanisms of CCEP are not yet fully understood. Thus, there is no consensus in measuring N1 or N2 responses to monitor brain function intraoperatively. The integrity of the language pathway was monitored using the maximum N1 amplitude in our previous studies (Yamao et al., 2014, 2017b), while N2 was adapted to monitor the language pathway in another intraoperative study (Saito et al., 2014). Additionally, because of a limited number of cases, a genuine cut-off value for application in intraoperative monitoring for preserving brain function is unclear. Fourth, the anesthetic effects on CCEP responses remains unclear. Intraoperative CCEPs were recorded in the awake surgery by intravenous propofol infusion. The depth of general anesthesia is usually monitored by the bispectral index, and in previous studies (Yamao et al., 2017a; Suzuki et al., 2019), even under deep anesthesia within a bispectral index monitor range of 40 ± 5, CCEPs were available. In previous cases (Jones et al., 2014; Yamao et al., 2017b), CCEPs could also be recorded under general anesthesia by using other intravenous anesthetics (ketamine or etomidate) or volatile anesthetics (sevoflurane or isoflurane). Although CCEPs are feasible under deep anesthesia, future systematic studies are required to clarify the stimulation protocol and anesthetic effects on the CCEPs. Fifth, we do not discuss which tracts from the CCEP connectivity are reflected in the CCEP responses. DES to the white matter pathway is required to detect the tracts beneath the sulci of the cortex or the lesion. Because DES may not be feasible in all cases due to clinical limitations, the combination of 50 Hz with 1 Hz subcortical electrical stimulation may help clarify the function of the CCEP connectivity.

## Conclusions

Many institutions, including our own, have recently reported the use of CCEPs for intraoperative mapping to preserve brain function, such as language and motor function. Although alternative preoperative neuroimaging studies, i.e., functional MRI or diffusion tractography, are still required, intraoperative CCEPs may allow efficient mapping and monitoring of functional networks. This single-pulse ES method may represent a new intraoperative monitoring technique to preserve language or motor function under general anesthesia only, analogous to MEPs or SEPs. We hope that further studies will establish intraoperative CCEP methods under general anesthesia.

## Author Contributions

YY: data collection, literature review, and manuscript writing. RM, TKi, and TKu: data collection and manuscript review. KY and SM: supervision. All authors contributed to the article and approved the submitted version.

## Conflict of Interest

The authors declare that the research was conducted in the absence of any commercial or financial relationships that could be construed as a potential conflict of interest.

## Abbreviations

AF: arcuate fasciculus
AL: anterior language area
CCEP: cortico-cortical evoked potential
DES: direct electrical stimulation
ES: electrical stimulation
FAT: frontal aslant tract
IFG: inferior frontal gyrus
IFOF: inferior fronto-occipital fasciculus
MEP: motor evoked potential
MRI: magnetic resonance imaging
PL: posterior language area
SEP: somatosensory evoked potential
SMA: supplementary motor area
UF: uncinate fasciculus.
